# Annotations

**Published:** 1887-05-14

**Authors:** 


### Annotations.

**Hospital Book-keeping.** The result of the prizes offered for the best essays and accounts of hospital housekeeping is disappointing. Every hospital of any importance received a copy of the special supplement, forms of accounts, and an invitation to compete for these prizes. In the result, however, few have accepted the invitation, and none appear to have understood that something more than the mere filling in of the return was required. In other words, no reports upon the working and housekeeping experiences of particular institutions, which can in any sense be called exhaustive, are forthcoming. In these circumstances it will be difficult, if not impossible, to award the prizes at all: a result which we very much regret, although it is doubtless due to the fact that heretofore individual managers and individual institutions have been so absorbed in their own particular work that they have, as a rule, refrained from studying the work of others. The feeling is no doubt growing that hospitals constitute an important department in the social economy of the day, but being of recent growth, the isolation of individual workers is still the rule and not the exception. One good result has, however, followed this movement on the part of The Hospitals Association, at the instigation of Mr. Cuthbert Quilter, many institutions having expressed their willingness to rearrange their accounts so as to include the items set forth in the return which was prepared in connection with this competition. As a consequence, most of the well-managed hospitals will henceforth prepare their accounts for publication on something like a common basis, so that the working of particular institutions may be easily and accurately compared for the future. Such a result is matter for congratulation, and cannot fail to be an encouragement to all who take a genuine interest in the welfare of our hospitals.

**Hospital Sunday.** It is a common saying that an Englishman never knows when he is beaten, and Mr. William Carnie, clerk and treasurer of the Aberdeen Royal Infirmary, is a striking example of the truth of this statement. Over and over again he has asserted that to his institution belongs the credit of establishing Hospital Sunday, because so long ago as 1763 it was resolved that a collection should be made for the infirmary in every parish within the bounds of the Church Synod on the first Sabbath of every year. Mr. Burdett, in his history of the Hospital Sunday and Saturday Funds, published by Kegan Paul and Co., has shown that in so acting the Aberdeen Infirmary did but follow the example of voluntary hospitals all over the country, the managers of which from the earliest days have caused collections to be made, in the churches and chapels in the districts to which they ministered, on behalf of their institutions. These local collections, however, established locally, and for one local institution only, have nothing whatever to do with the Hospital Sunday movement. Hospital Sunday is an organisation, established originally in Birmingham, which unites the people of all religious denominations on a given day, who collectively contribute, not to one, but to all the medical institutions, either in turn or each year, by means of collections held in the different places of worship. This is a far different thing from a collection or collections made in certain places of worship for a particular charity, either on the same day or on different days year by year. The local church collections were not confined to Aberdeen, but were held all over the country for particular institutions in particular localities. For this reason they excited only a local interest, and though undoubtedly useful to local charities, they failed to attain a national importance, much less an international interest and imitation, as Hospital Sunday has done in the thirty years since it was first established by Canon Miller at Birmingham.

**Dynamite as a Life Preserver.** Dynamite and nitro-glycerine are so generally regarded as dangerous from their life-destroying properties, that it may be well to place on record the fact that they may, in certain circumstances, be used to preserve life. The *Sei-i-Kwai*, the medical journal of Japan, records a case of the restoration of a woman, apparently dead, by the hypodermic injection of nitro-glycerine. The patient, a woman, sank and collapsed, and life was believed to be quite extinct. All the usual remedies were administered without success, and the case was regarded as hopeless by the medical attendant, Dr. M. H. Sackersteer, when he bethought himself to try nitro-glycerine as a last resort. He injected ten drops of a solution, and after a minute's interval the patient sighed, and in five minutes the pulse was felt and the heart was distinctly heard. Subsequently a flush came over the face, the eyes opened, and the muscles, which had assumed the rigidity of death, gradually relaxed, when the patient became conscious. Within a few days a complete recovery was effected. Under such circumstances it may be well to mention that Martindale, the eminent chemist, gives several forms for the administration of nitro-glycerine in solution, hypodermically, and by drops, tablets, and troches, the latter of which are attractive in appearance and cannot be distinguished by the patient from ordinary chocolate creams. We have seen some startling effects of the administration of this remedy in the out-patient department, and no doubt, in extreme cases, the medical profession will find nitro-glycerine a friend in need where all other remedies have failed.

